# Pre-hospital plasma transfusion: a valuable coagulation support or an expensive fluid therapy?

**DOI:** 10.1186/s13054-019-2524-4

**Published:** 2019-07-01

**Authors:** Christian Fenger-Eriksen, Dietmar Fries, Jean-Stephane David, Pierre Bouzat, Marcus Daniel Lance, Oliver Grottke, Donat R. Spahn, Herbert Schoechl, Marc Maegele

**Affiliations:** 1 Department of Anaesthesiology, Aarhus University Hospital, Aarhus, Denmark; 2 Department for General and Surgical Critical Care Medicine, Medical University Innsbruck, Innsbruck, Austria; 3 Department of Anesthesiology and Critical Care Medicine, Lyon Sud Hospital and Claude Bernard University, Lyon Est School of Medicine, Lyon, France; 4 Grenoble Alps Trauma Center, Department of Anesthesiology and Intensive Care Medicine, Grenoble University Hospital, Grenoble, France; 5 Weill-Cornell Medicine-Qatar, Department of Anesthesiology, ICU & Perioperative Medicine, Hamad Medical Corporation, Doha, Qatar; 6 Department of Anesthesiology, RWTH Aachen University Hospital, Aachen, Germany; 7 Institute of Anesthesiology, University and University Hospital of Zurich, Zurich, Switzerland; 8 Department of Anesthesiology and Intensive Care Medicine, AUVA Trauma Centre Salzburg, Academic Teaching Hospital of the Paracelsus Medical University, Salzburg, Austria; 9 Ludwig Boltzmann Institute for Experimental and Clinical Traumatology, Vienna, Austria; 10 Department of Traumatology and Orthopedic Surgery, Cologne-Merheim Medical Center, Institute for Research in Operative Medicine (IFOM), University Witten-Herdecke, Cologne, Germany

Two recent clinical trials with conflicting results have refuelled the discussion on pre-hospital plasma in trauma. The multicentre, cluster-randomized PAMPer trial assessed the efficacy and safety of two units of pre-hospital plasma versus standard care without plasma in 501 trauma patients at risk for haemorrhagic shock during air medical transport to a designated US trauma centre [1]. The mortality at 30 days was lower in the plasma compared to the standard care group (23% vs 33%; *p* = 0.03). The randomized, placebo-controlled COMBAT trial compared the same plasma volume versus isotonic saline in 144 haemorrhagic shocked trauma patients within a US ground EMS and a single US trauma centre but mortality at 28 days did not differ between trial groups (15% vs 10%; n.s.) [2]. Table 1 summarizes the basic characteristics of both trials. The results from both trials need to be viewed with caution against their limitations and may not be translated directly into routine without addressing a number of critical issues.

**Table 1:** Basic characteristics of both trials

	COMBAT		PAMPer	
	FFP	Standard	FFP	Standard
Setting	US ground EMS transport (Denver) single-centre		US air EMS transport multicentre	
Randomisation	Individual randomisation by content of cooling boxes; staff non-blinded		Cluster randomisation at monthly intervals; staff non-blinded	
Inclusion criteria	BP < 70 mmHg *or* BP 71–90 mmHg + HR > 108/min		BP < 70 mmHg *or* BP < 90 mmHg and HR > 108/min	
Patients included (*n*)	65 vs 60		230 vs 271	
Age median (IQR)	33 (25–51)	33 (25–42)	44 (31–59)	46 (28–60)
Male (%)	80	85	71	74
Blunt injury (%)	46	53	81	73
Injury severity Score median (IQR)*	27 (10–41)	27 (11–36)	22 (14–33)	21 (12–29)
Prothrombin time ratio or INR on hospital arrival	1.3	1.2	1.2	1.3
Pre-hospital management				
Pre-hospital intubation (%)	Not provided	Not provided	50	50
Pre-hospital RBCs (%)	Not provided	Not provided	26	42
Pre-hospital crystalloids (mls) median (IQR)	150 (0–300)	250 (100–500)	500 (0–1250)	900 (0–1500)
Tranexamic acid within 6 h (%)	9	13	Not provided	Not provided
Intervention	2 U pre-thawed FFP up to 5d old FFP vs standard		2 U apheresis FFP (approx. 500 ml) vs standard	
Median Transportation time median (IQR)	28 (22–34) min	24 (19–31) min	42 (34–53) min	40 (33–41) min
Outcome				
Primary endpoint	Mortality 28 days		Mortality 30 days	
Mortality 28/30d (%)	15	10	23	33
Mortality 24 h (%)	12	10	14	22

*Combat trial New Injury Severity Score was used

A single drop in blood pressure as an inclusion criterion for both trials is problematic as pre-hospital hypotensive episodes can have non-bleeding reasons (e.g. anaesthesia, cardiac, spinal trauma or wrong readings), and, in PAMPer, half of the patients had received pre-hospital intubation/mechanical ventilation while for COMBAT no details were provided. Both trials aimed for patients “at risk for haemorrhagic shock” or “thought to be due to acute blood loss” but no signs of bleeding were considered for inclusion. Notably, 111 patients in PAMPer had received unspecified pre-treatment prior to inclusion which may have introduced bias. The time span for inclusion expanded over 3 years with trauma care subject to change over time, e.g. the increasing widespread use of antifibrinolytic tranexamic acid (TXA). In COMBAT, 10% of patients had received TXA while its use was not reported for PAMPer.

There was high mortality difference at 24-h and 28/30-days within both control arms which had assumingly received comparable US standard trauma care after hospital admission (Table 1). With identical entry criteria, this difference may only be explained by differences in injury severity, volume status and further pattern and/or patient care; but no specific details were provided. The comparison of injury severity between both trials is difficult due to different scores applied. However, the mortality in PAMPer was higher than in COMBAT and that reported elsewhere which limits the external validity of findings. In the European RETIC trial on early coagulation factor concentrates versus FFP in trauma the 30-day mortality was only 7.4% despite an ISS of 34 [3]. The German Trauma Registry (TR-DGU) confirms a mortality < 10% for an ISS 20–23 [4]. In PAMPer, there was no clinical benefit for plasma on the sequalae of hypovolaemic-haemorrhagic shock as 32% versus 29% of patients died in haemorrhagic shock.

The underlying mechanism by which the two units of pre-hospital plasma may have promoted lower mortality in PAMPer remains speculative. In both trials, no relevant improvements in standard/viscoelastic coagulation assays were reported after pre-hospital plasma. A statistically relevant but clinically insignificant shorter prothrombin ratio was reported for the plasma group (1.2 vs 1.3) but cannot account for the observed difference in mortality. In COMBAT, more patients in the plasma group had an INR > 1.3. The INR quantifies only pro-coagulants and does not mirror concentrations of inhibitors. In trauma, INR can be prolonged despite upregulated thrombin generation potential [5]. Moreover, the INR of FFP is 1.3 [6]. Any beneficial effect of plasma to correct slightly elevated INR is futile and plasma has primarily an effect on coagulation parameters with extended volumes and performs best in patients bleeding and coagulopathic. The negative effects of plasma need to be considered [7]; in COMBAT a trend towards higher MOF was observed in the plasma group (6% vs 2%).

A large proportion of patients did not receive massive transfusion and most arrived at the hospital with the absence of clinical/laboratory signs of relevant coagulopathy. Furthermore, the number of pRBCs transfused in both trials was low and without any statistical difference. Notably, half of the PAMPer patients had received pre-hospital pRBCs. Moreover, no difference in mortality in massively transfused patients independent of pre-hospital plasma administration was reported. Accordingly, only patients with less severe injury would have benefited from pre-hospital plasma. In PAMPer, 42% of the standard care patients had received pre-hospital pRBCs (26% in the FFP group) and almost twice as much crystalloids prior to hospital admission. Both measures could have contributed to the higher mortality in the standard care group in PAMPer. The transfusion trigger, however, remains unknown. For COMBAT, the authors admitted that median fibrinogen levels and other coagulation factors on hospital arrival were within reference ranges; and slightly higher in the standard of care non-FFP group.

As demonstrated, the pre-hospital administration of plasma to trauma patients is technically/logistically feasible both in air and ground EMS. However, the different conclusions of the trials leave the question unsolved to whether pre-hospital plasma may be of any clinical benefit for the target population. As PAMPer patients had received pre-hospital pRBCs and more intravenous fluids a simple “volume” effect cannot be excluded. Risk-benefits need to be balanced against other challenges, e.g. infrastructure, logistics and costs, and an early goal-directed approach using coagulation factor concentrates (e.g. fibrinogen, which is critically depleted first during bleeding [8]), TXA, and permissive hypotension along with surgical bleeding control to limit further blood loss and stabilize coagulation function pre-hospital may be an alternative as outlined in the updated European trauma guideline [9]. Protective effects to the glycocalyx and endothelial barrier integrity have been linked to the fibrinogen component rather than to plasma per se [10]. A median 3.8 g fibrinogen concentrate can increase clot stability by 5.2 mm at 5 min of viscoelastic test initiation while TXA can decrease lysis by 5.4% [11].

From a European perspective, the blind pre-hospital administration of plasma to potentially non-coagulopathic patients with short transportation times cannot be justified. More clearly defined studies are necessary to justify logistics and costs associated with pre-hospital blood product transfusion.

## Data Availability

Not applicable.

## Abbreviations

ARDS: Acute respiratory distress syndrome
bpm: Beats per minute
COMBAT: Plasma-first Resuscitation to Treat Haemorrhagic Shock during Emergency Ground Transportation in an Urban Area: A Randomised Trial
CPP: Cerebral perfusion pressure
CRASH-2: Clinical Randomization of an Antifibrinolytic in Significant Hemorrhage 2 Trial
EMS: Emergency Medical Service
FFP: Fresh frozen plasma
INR: International Normalized Ratio
ISS: Injury Severity Score
MOF: Multiorgan failure
n.s.: Non-significant
PAMPer: Prehospital Plasma during Air Medical Transport in Trauma Patients at Risk for Haemorrhagic Shock Trial
pRBC: Packed red blood cells
PTr: Prothrombin ratio
RETIC: Reversal of Trauma-induced Coagulopathy using First-line Coagulation Factor Concentrates or Fresh Frozen Plasma Study
TBI: Traumatic brain injury
TR-DGU: TraumaRegistry of the Deutsche Gesellschaft für Unfallchirurgie
TXA: Tranexamic acid
US: United States
